# Lack of evidence of disease contamination in ovarian tissue harvested for cryopreservation from patients with Hodgkin lymphoma and analysis of factors predictive of oocyte yield

**DOI:** 10.1038/sj.bjc.6603050

**Published:** 2006-03-28

**Authors:** T Seshadri, D Gook, S Lade, A Spencer, A Grigg, K Tiedemann, J McKendrick, P Mitchell, C Stern, J F Seymour

**Affiliations:** 1 Monash Medical Centre, Clayton, Victoria 3168, Australia; 2 Royal Women's Hospital, and Melbourne IVF, East Melbourne, Victoria 3002, Australia; 3 The Peter MacCallum Cancer Centre, Department of Haematology and Medical Oncology Level 5, Locked Bag 1, A’Beckett st, East Melbourne, Victoria 8006, Australia; 4 Alfred Hospital, Prahran, Victoria 3181, Australia; 5 Royal Melbourne Hospital, Parkville, Victoria 3052, Australia; 6 Royal Children's Hospital, Parkville, Victoria 3052, Australia; 7 Box Hill Hospital, Box Hill, Victoria 3128, Australia; 8 Austin Hospital, Heidelberg, Melbourne, Victoria, 3084, Australia

**Keywords:** fertility, Hodgkin lymphoma, ovarian cryopreservation

## Abstract

Ovarian cryopreservation is a promising technique to preserve fertility in women with Hodgkin lymphoma (HL) treated with chemotherapy. Thus, the aim of this study was to examine harvested ovarian tissue for subclinical involvement by HL by morphology/immunohistochemistry, and to define patient and treatment factors predictive of oocyte yield. This was a retrospective analysis of 26 ovarian tissue samples harvested for cryopreservation from women with HL. Histology, immunohistochemistry and follicle density (number mm^−3^) was examined. Disease status and preharvest chemotherapy details were obtained on 24 patients. The median age was 22 years (range 13–29). Seven of 24 patients had infradiaphragmatic disease at time of harvest. Nine of 20 patients had received chemotherapy preharvest (ABVD (Adriamycin®, Bleomycin, Vinblastine and Dacarbazine)=7, other regimens=2). The seven receiving ABVD showed no difference in follicle density compared to patients not receiving treatment (*n*=14); (median=1555 *vs* 1620 mm^3^ *P*=0.97). Follicle density measurement showed no correlation with patient age (*R*^2^=0.0001, *P*=0.99). There was no evidence of HL involvement in the 26 samples examined (95% CI=0–11%). In conclusion, subclinical involvement of HL has not been identified in ovarian tissue, even when patients have infradiaphragmatic disease. Furthermore, the quality of tissue harvested does not appear to be adversely affected by patient's age or prior ABVD chemotherapy.

As Hodgkin lymphoma (HL) is a common malignancy in young people (Blumenfeld *et al*, 2002), fertility preservation is an important issue to address when counselling patients prior to chemotherapy. With current regimens of chemotherapy and radiotherapy a large proportion of patients will be cured of their illness, but with the risk of long-term toxicity, particularly with recently developed intensive chemotherapy programmes such as escalated-dose BEACOPP (Bleomycin, Etoposide, Adriamycin®, Cyclophosphamide, Oncovin®, Procarbazine, Prednisolone) (Diehl *et al*, 2003).

A major concern for female patients is the risk of long-term infertility and premature ovarian failure. Depending on the chemotherapy regimen used this risk can range from very low (0–10%) with ABVD (Adriamycin®, Bleomycin, Vinblastine and Dacarbazine) (Seymour, 2001) to high (∼50%) with BEACOPP (Behringer *et al*, 2004) and very high for those who require bone marrow transplantation (Mertens *et al*, 1998; Watson *et al*, 1999; Grigg, 2004).

Unlike sperm cryopreservation, oocyte preservation is invasive and burdensome for patients and has so far had limited applicability in humans. Embryo cryopreservation has a pregnancy rate of 15–25%, but as considerable time is required to obtain mature follicles chemotherapy administration may be delayed with potential detrimental effect on patient outcome. Moreover, this option requires donor sperm, often not available to women without a stable partner, which will often be the case among adolescents and young adult women (Wood *et al*, 1997).

Ovarian cryopreservation is a promising strategy for preserving fertility in these patients. Harvesting of ovarian tissue via laparoscopic surgery can be performed at any time during the menstrual cycle. Furthermore, the removal and appropriate cryopreservation of a small area of ovary containing an abundance of primordial follicles has more theoretical potential for future fertility than cryopreserved oocytes (Gook *et al*, 1999, 2003). This tissue can then be reimplanted either orthotopically (Radford *et al*, 2001; Meirow *et al*, 2005), or heterotopically (Donnez *et al*, 2004; Oktay *et al*, 2004; Wolner-Hanssen *et al*, 2005), with the demonstrated capacity to restore both endocrine function and fertility (Donnez *et al*, 2004; Meirow *et al*, 2005).

The data on the success of this procedure is largely limited to animal studies and case reports. It has been demonstrated that murine recipients of cryopreserved ovaries have had successful pregnancies (Gunasena *et al*, 1997; Shaw *et al*, 2000). Autotransplantation using fresh ovarian tissue has been successful in reinstating monkeys’ ovarian cycles and producing oocytes resulting in pregnancy with subsequent live birth (Lee *et al*, 2004).

In humans, there has been a report of a temporary restoration of endocrine function after cryopreserved ovarian tissue was auto-transplanted back into a patient with HL (Radford *et al*, 2001). In addition, there have been two reports of a successful pregnancy and live birth, which resulted from the reimplantation of frozen ovarian tissue into patients with HL and non-Hodgkin's lymphoma (NHL) (Donnez *et al*, 2004; Meirow *et al*, 2005).

One of the potential concerns with this procedure is the risk of disease reimplantation. Lymphoma involving the ovary is indeed rare. However, there has been at least one case report of clinically manifest HL involving the ovary (Khan *et al*, 1986) and a report of HL resulting in omental caking (Jacobs *et al*, 1996). Thus, there is the potential for occult ovarian involvement at the time of tissue harvesting. There are only two reports of histological assessment of cryopreserved ovarian tissue in patients with HL and neither of these found any evidence of HL involvement of the harvested tissue by light microscopy (Meirow *et al*, 1998; Kim *et al*, 2001). Kim *et al* (2001) xenotransplanted ovarian tissue from women with HL into immunodeficient mice and found no evidence of disease transmission, although the capacity of primary HL cells to grow in such a xenograft model is unknown. Nevertheless, a murine study has shown the capacity of reimplanted syngeneic ovarian tissue to transmit lymphoma in six out of seven cases, however, this study was performed using a high grade NHL (Shaw *et al*, 1996).

Another difficulty is identifying patients where the harvested tissue will contain adequate numbers of follicles. A single case report suggested that tissue collected after previous chemotherapy may contain marginal numbers of follicles with subsequent transient functional capacity after reimplantation (Radford *et al*, 2001). There have been no prior studies exploring factors associated with the adequacy of follicle numbers in harvested ovarian tissue.

The aim of this study was to examine the ovarian tissue harvested from a cohort of young women with HL for subclinical involvement with lymphoma using sensitive and specific immunohistochemistry. We also aimed to define patient and treatment factors that may play a role in the quality of the tissue harvested.

## MATERIALS AND METHODS

### Patients

This was a retrospective analysis of ovarian tissue samples obtained from 26 women with HL aged between 13 and 29 years who had undergone ovarian tissue harvest at laparoscopy between December 1995 and August 2005. All patients were referred to Reproductive Services/Melbourne IVF, Royal Women's Hospital for fertility preservation by their treating oncologist. The point in treatment when this tissue was obtained was left to the discretion of the treating physician and the patient. Consent was obtained for both the laparoscopic removal and storage of ovarian tissue and histological examination.

Details of the patients’ disease and treatment were obtained from the treating physicians’ medical records via a questionnaire completed by the treating physician. Preharvest patient and treatment characteristics were subsequently correlated with the ovarian histology and follicle density. Data regarding premature ovarian failure in the cohort following HL treatment was not collated.

### Ovarian tissue cryopreservation

A wedge section of ovary (surface area 1–6 cm^2^) was removed in all but two patients in whom a whole ovary was removed. A small area was fixed immediately in 10% formalin for histological and immunohistochemical analysis. The remaining tissue was trimmed to approximately 1 mm thick, removing all medulla and subsequently cut into small slices (∼2 × 4 mm). The cryopreservation procedure has been previously described in detail (Gook *et al*, 1999). Briefly, the slices were soaked in cryoprotectant (1.5 mol l^−1^ propanediol and 0.1 mol l^−1^ sucrose in phosphate buffered saline) for 90 min, followed by cooling in a control rate freezing machine to −150°C and stored in liquid nitrogen vapour.

### Follicle density and immunohistochemistry

Fixed tissue was processed and embedded in paraffin wax. Histological sections, stained with haematoxylin and eosin, were screened for ovarian follicles and the presence of the distinctive malignant Reed–Sternberg (RS) cells characteristic of HL. A minimum of 10 sections selected randomly throughout the fixed piece of ovarian tissue were examined to determine follicle density (number per cubic mm).

Additional sections were examined for the presence of malignant cells expressing the surface markers CD15 and CD30 characteristic of HL. These two markers were chosen as RS cells are positive for CD30 in up to 100% of cases (Stein *et al*, 1985) and the incidence of CD15 positivity is very high at 75–85% (Jaffe *et al*, 2001). Thus, a minimum of three random sections per patient of both ovarian cortex and medulla were examined histologically for involvement with HL by a single independent expert pathologist.

Following heat antigen retrieval for 2 min in 10 mmol l^−1^ citrate buffer pH 6.0 at 125°C, sections were incubated with primary antibody (CD15 – 1 in 50 dilution of clone MMA, Becton Dickinson, Franklin Lakes, New Jersey, USA; CD30 – 1 in 400 dilution of clone Ber-H2, Dakocytomation) for 30 min at room temperature. Sections were washed and incubated with a second antibody conjugated to a horseradish peroxidase DAB detection system (Mouse Envision, Dakocytomation).

All statistical analyses were performed using Minitab software.

## RESULTS

Data regarding patient's disease status were available for 24 of the 26 patients and are outlined in Table 1.

Nine patients had received chemotherapy prior to harvest with seven patients receiving ABVD, one received Stanford V (Horning *et al*, 2002) and one ChlVPP (chlorambucil, vinblastine, procarbazine, prednisolone), etoposide/vincristine/epirubicin. The median number of ABVD cycles received prior to harvest was 6 (range 2–6). The median time between the final cycle of ABVD chemotherapy and ovarian harvest was 2 months (range 2–24 months).

At diagnosis of HL nine patients had stage III or stage IV disease and at the time of harvest seven patients had disease below the diaphragm. Of these seven patients five had intra-abdominal disease, one had bony disease in the femur and in one patient the exact site(s) of infradiaphragmatic disease at the time of harvest were not recorded.

There was no evidence of HL involvement by morphology or immunohistochemistry in any of the 26 samples examined (95% CI for ‘true’ rate of involvement=0–11%). No inflammatory infiltrate or any other atypical findings were noted in the samples examined.

The range of follicle densities was 45–4512 follicles mm^−3^ for the 26 patients. The seven patients who had received ABVD chemotherapy preharvest showed no statistical difference in follicle density compared to patients not receiving treatment (*n*=14) (median number of follicles for those receiving ABVD was 1555 mm^3^ (range 45–4512 mm^3^) *vs* 1620 mm^3^, (range 459–2840 mm^3^) for those not receiving prior treatment, *P*=0.97). Follicle density measurement for the entire cohort and for the 14 untreated patients showed no correlation with patient age (*R*^2^=0.0001, *P*=0.99 and *R*^2^=0.011, *P*=0.93, respectively).

## DISCUSSION

With technology continually improving for cryopreservation and now with successful pregnancies from this technique, the safety and timing of ovarian harvesting are important issues to address.

We did not identify any histological evidence of lymphomatous involvement in 26 patients with HL undergoing ovarian harvesting despite some patients having definite intra-abdominal disease at the time of ovarian collection. While we cannot exclude the possibility of disease involvement in all cases, our data suggest the frequency is likely to be very low. Possible risk factors for involvement may be predicted to be intra-abdominal disease and the presence of B symptoms at the time of collection as these features imply more aggressive disease behaviour. Studies of ‘high risk’ patients are needed to better define the risk of ovarian involvement in these specific settings.

One of the main hurdles to overcome with ovarian cryopreservation is the loss of primordial follicles due to ischaemia prior to revascularisation. Thus, optimal follicle yield is of paramount importance. Although follicle density was determined from a single biopsy and distribution varies throughout the ovary, the follicle densities for the HL patients are similar to those observed for a group of young women with other types of cancer (Gook *et al*, 2005). Whether a single biopsy of an ovary is adequate to exclude HL involvement of the entire ovary is uncertain and one should consider random examination of several slices within the biopsy specimen prior to autotransplantation of the remaining tissue.

In this study, we used immunohistochemistry in addition to morphology to identify RS cells, as it has been shown to be very sensitive (Stein *et al*, 1985; Jaffe *et al*, 2001). One must note that RS cells are very fragile and are easily damaged by some cell manipulation procedures (Irsch *et al*, 1998). Thus, although there is no data available, it is possible that the cryopreservation process may selectively damage RS cells, thus making examination of poststorage tissue less sensitive. However, our histological analysis was performed on tissue prior to the cryopreservation process. Furthermore, RS cells are generally associated with a rich inflammatory cell background and this was not evident in the ovarian sections examined. One additional point to note is that a number of tumours have recently been shown to be derived from a small population of ‘cancer stem cells’ (Zhang and Rosen, 2006). It is currently unknown whether such a stem-cell pool for RS cells exists, but if this were the case, it is unclear whether the immunohistochemical screen used here would detect these rare cells putatively able to transfer the tumour.

This paper also addresses what factors may influence the quality of the ovarian tissue harvested. Not surprisingly given the young age of patients with HL analysed in our series (maximum 29 years), age did not adversely affect the quality of the tissue harvested. Furthermore, we also found no difference in the quality of tissue harvested between those patients who had prior ABVD chemotherapy and those who had no prior treatment. This is relevant given that a large proportion of patients are cured of their disease with ABVD chemotherapy with the retention of their fertility. Thus, we do not recommend routine collection prior to ABVD chemotherapy, but rather at relapse prior to salvage therapy or at primary diagnosis if intensified therapy (such as escalated BEACOPP) is planned. This is in contrast to the Edinburgh criteria recently published (Wallace *et al*, 2005), which recommend that only patients who have not received chemotherapy be selected as candidates for ovarian cryopreservation. Given the considerable costs involved and the need for invasive surgery this recommendation may need to be modified. We do note, however, that in our study the median number of cycles of ABVD chemotherapy administered was six and it is possible that more cycles of chemotherapy could be associated with reduced follicle density on harvest. Furthermore, even though ABVD is generally viewed as having a very low risk for sterility, older patients receiving numerous cycles of ABVD are more likely to suffer premature infertility and thus may benefit from ovarian harvest prior to treatment. It is unsure whether prior chemotherapy will influence the ability of the graft to revascularise once it has been transplanted back into the host or whether many of the follicles are atretic – a characteristic that is difficult to determine from light microscopy alone – (Familiari *et al*, 1993) and further studies are required to explore these issues. None of our patients have had their ovarian tissue reimplanted thus the *in vivo* characteristics of the harvested tissue is not known.

In conclusion, ovarian harvesting appears to be a feasible consideration in young female patients with HL even in those with intra-abdominal involvement and that prior chemotherapy with nonsterilising regimes such as ABVD does not adversely influence the follicle yield.

## CONTRIBUTIONS

T.Seshadri drafted the article and collated and analysed the data; D Gook drafted the Materials and methods sections and developed the ovarian tissue cryopreservation procedure and assessed follicle density and assisted with data collection; C Stern coordinated and harvested the ovarian tissue; S Lade performed histological and immunohistochemical analysis for HL. A Spencer, A Grigg, K Tiedemann, J McKendrick, P Mitchell provided patient data and reviewed the manuscript. J Seymour provided original thought and ideas, assisted with drafting and provided all statistical analyses.

## Figures and Tables

**Table 1:** Patient characteristics

*Age*	*Years*
Median (year)	22; range (13–29)
	
*Histological subtype*	*n* (%)
Nodular sclerosing	23 (96)
Mixed cellularity	1 (4)
	
*Stage at diagnosis*	*n* (%)
I/II	15 (63)
III/IV	9 (37)
	
*B symptoms at diagnosis*	*n* (%)
Yes	9 (37)
No	15 (63)
	
*Immunophenotype*	*n*
CD30+	6
CD 15+	5
Not performed	16
	
*Disease sites at time of harvest*	*n* (%)
Disease below diaphragm	7 (29)
Disease above diaphragm only	16 (67)
Unknown	1 (4)
	
*Prior treatment*	*n* (%)
Nil	14 (58)
ABVD	7 (29)
Other chemotherapy	2 (8)
Radiotherapy to abdomen	2 (8)
